# Isolation of *Pediococcus acidilactici* Kp10 with ability to secrete bacteriocin-like inhibitory substance from milk products for applications in food industry

**DOI:** 10.1186/1471-2180-12-260

**Published:** 2012-11-15

**Authors:** Sahar Abbasiliasi, Joo Shun Tan, Tengku Azmi Tengku Ibrahim, Ramakrishnan Nagasundara Ramanan, Faezeh Vakhshiteh, Shuhaimi Mustafa, Tau Chuan Ling, Raha Abdul Rahim, Arbakariya B Ariff

**Affiliations:** 1 Department of Bioprocess Technology, Faculty of Biotechnology and Biomolecular Sciences, Universiti Putra Malaysia, Serdang, Selangor, 43400 UPM, Malaysia; 2 Department of Veterinary Preclinical Sciences, Faculty of Veterinary Medicine, Universiti Putra Malaysia, Serdang, Selangor, 43400 UPM, Malaysia; 3 Chemical and Sustainable Process Engineering Research Group, School of Engineering, Monash University, Bandar Sunway, Selangor, 46150, Malaysia; 4 Institute of Bioscience, Universiti Putra Malaysia, Serdang, Selangor, 43300, Malaysia; 5 Department of Microbiology, Faculty of Biotechnology and Biomolecular Sciences, Universiti Putra Malaysia, Serdang, Selangor, 43400 UPM, Malaysia; 6 Institute of Biological Sciences, Faculty of Science, Universiti Malaya, Lembah Pantai, Kuala Lumpur, 50603, Malaysia; 7 Department of Cell and Molecular Biology, Faculty of Biotechnology and Biomolecular Sciences, Universiti Putra Malaysia, Serdang, Selangor, 43400 UPM, Malaysia

**Keywords:** Lactic acid bacteria, *Pediococcus acidilactici*, Bacteriocin-like inhibitory substance, *Listeria monocytogenes*, Fermentation, Identification

## Abstract

**Background:**

Lactic acid bacteria (LAB) can be isolated from traditional milk products. LAB that secrete substances that inhibit pathogenic bacteria and are resistant to acid, bile, and pepsin but not vancomycin may have potential in food applications.

**Results:**

LAB isolated from a range of traditional fermented products were screened for the production of bacteriocin-like inhibitory substances. A total of 222 LAB strains were isolated from fermented milk products in the form of fresh curds, dried curds, and ghara (a traditional flavor enhancer prepared from whey), and fermented cocoa bean. Eleven LAB isolates that produced antimicrobial substances were identified as *Lactococcus lactis*, *Lactobacillus plantarum*, and *Pediococcus acidilactici* strains by biochemical methods and 16S rDNA gene sequencing. Of these, the cell-free supernatant of Kp10 (*P. acidilactici*) most strongly inhibited *Listeria monocytogenes*. Further analysis identified the antimicrobial substance produced by Kp10 as proteinaceous in nature and active over a wide pH range. Kp10 (*P. acidilactici*) was found to be catalase-negative, able to produce β-galactosidase, resistant to bile salts (0.3%) and acidic conditions (pH 3), and susceptible to most antibiotics.

**Conclusion:**

Traditionally prepared fermented milk products are good sources of LAB with characteristics suitable for industrial applications. The isolate Kp10 (*P. acidilactici*) shows potential for the production of probiotic and functional foods.

## Background

The screening of microorganisms isolated from naturally occurring processes is the most common method of obtaining strains useful for industrial applications. This holds true for lactic acid bacteria (LAB), which are used worldwide to produce a variety of fermented foods [1]. Because LAB have been used in food production for centuries without posing any health risks, they are designated as generally regarded as safe (GRAS) microorganisms [2].

LAB are normally found in nutrient-rich environments and are able to grow in most raw foods. These bacteria are fastidious and require fermentable carbohydrates, amino acids, fatty acids, salts, and vitamins for growth [3]. Because of their metabolic properties, LAB play an important role in the food industry, contributing significantly to flavor, texture, and frequently the nutritional value of foods [4].

Because of the rapid rise and spread of multi-resistant bacterial pathogens, new methods are needed to combat infection. Antibiotics are widely used to prevent the spread of pathogenic bacteria; however, many antibiotics are broad-spectrum drugs that kill bacterial species indiscriminately [5]. Bacteriocins have a relatively narrow spectrum of killing activity, and some can be considered pathogen-specific designer drugs. Given the diversity of bacteriocins produced in nature, it may be a relatively simple task to identify bacteriocins effective against specific human pathogens [5]. In addition, bacteriocin use may reduce the need for chemical additives in food and minimize the intensity of food processing techniques, contributing to the production of more healthful foods [6].

In recent years, attention has been focused on LAB from different sources that produce bacteriocins that are considered safe as food biopreservatives and can be degraded by gastrointestinal proteases [7]. These probiotic compounds have been used in a variety of industrial applications relevant to both human and animal health without producing side effects. There is an ongoing need to identify new strains with useful characteristics. Therefore, the main objective of this study was to isolate and characterize LAB that produce bacteriocin-like inhibitory substances (BLIS) from traditionally prepared milk products (e.g., fresh curds, dried curds, and ghara) and locally fermented cocoa beans. These fermented products do not use starter cultures; fermentation is the result of wild flora present in the surrounding environment. Wild LAB strains represent a natural reservoir of strains not exposed to any industrial selection and are potential probiotics and bacteriocin producers [8]. In this study we identified and characterized LAB strains that produce high BLIS levels for possible applications in the food industry.

## Results

### Isolation of BLIS-producing strains

A total of 222 LAB strains were isolated from nine test samples (Table 1). After preliminary identification, 11 of these strains were found to produce antimicrobial substances. All 11 isolates were gram-positive, catalase-negative, and able to ferment glucose and produce acid. Both cocci and bacilli were identified. The isolates Kp8 and Kp10 showed the highest antimicrobial activity (888.56 AU/mL).

**Table 1:** Morphological, biochemical characteristics and antimicrobial activity of LAB isolates

	**Fresh curds**					**Dried curds**					**Ghara**	**Fermented cocoa beans**			
	**Pg**	**Cam**		**Pak**		**Ky**	**Kp**		**Sat**	**Kbo**	**Gh1**	**C**			
		**Cam4**	**Cam5**	**Pak1**	**Pak7**		**Kp8**	**Kp10**				**C6**	**C7**	**C13**	**C22**
No. of LAB isolates (cultured in MRS and M17)	10											8		26		20	20		40	40	10	48			
No. of isolates showing antimicrobial activity	0	2		2		0	2		0	0	1	4								
Cell morphology	ND	Bacilli		Bacilli		ND	Cocci		ND	ND	Cocci	Bacilli	Bacilli	Cocci	Cocci					
Gram stain reaction	ND	+		+		ND	+		ND	ND	+	+								
Catalase activity	ND	-		-		ND	-		ND	ND	-	-								
Glucose fermentation	ND	+		+		ND	+		ND	ND	+	+								
Activity (AU/mL) against *L. monocytogenes* ATCC15313	**ND**	**276.51**^**c**^	**276.51**^**c**^	**26.78**^**a**^	**26.78**^**a**^	**ND**	**888.56**^**d**^	**888.56**^**d**^	**ND**	**ND**	**115.21**^**b**^	**26.78**^**a**^	**26.78**^**a**^	**26.78**^**a**^	**26.78**^**a**^					

Positive reaction (+), negative reaction (−), not detected (ND).

Values with different superscript letters (a, b, c, d) are significantly different.

### Characterization of isolates with API 50 CHL

The carbohydrate fermentation patterns of the 11 isolates were determined by using the API 50 CHL micro-identification system (Table 2). The isolates Gh1, C22, and C13 were able to hydrolyze ribose, d-xylose, galactose, glucose, fructose, mannose, n-acetyl-glucosamine, amygdalin, esculin, arbutin, salicin, cellobiose, maltose, lactose, trehalose, starch, gentiobiose, and gluconate. However, mannitol and sucrose were hydrolyzed by Gh1 but not by C22 or C13. The isolates Kp8 and Kp10 were able to hydrolyze glycerol, l-arabinose, ribose, d-xylose, galactose, glucose, fructose, mannose, mannitol, n-acetyl-glucosamine, esculin, salicin, cellobiose, gentiobiose, and d-tagatose. The isolates Com4, Pak1, Com5, C6, C7, and Pak7 were able to hydrolyze, ribose, galactose, glucose, fructose, mannose, mannitol, n-acetyl-glucosamine, amygdalin, arbutin, esculin, salicin, cellobiose, maltose, lactose, melibiose, sucrose, trehalose, melezitose, and gentiobiose but differed in their ability to metabolize glycerol, sorbose, rhamnose, sorbitol, α-methyl-d-mannoside, α-methyl-d-glucoside, raffinose, turanose, d-tagatose, l-fucose, d-arabitol, and gluconate. To identify the isolates, their carbohydrate metabolism patterns were analyzed using the API database (Table 3).

**Table 2:** Biochemical profiles of LAB isolates

**LAB isolates**																									
		**Gh1**	**Com4**	**Pak1**	**Kp8**	**Com5**	**Kp10**	**C22**	**C6**	**C7**	**C13**	**Pak7**			**Gh1**	**Com4**	**Pak1**	**Kp8**	**Com5**	**Kp10**	**C22**	**C6**	**C7**	**C13**	**Pak7**
0	Control	-	-	-	-	-	-	-	-	-	-	-													
1	Glycerol	-	+	+	+	+	+	-	-	+	-	+	26	Salicin	+	+	+	+	+	+	+	+	+	+	+
2	Erythritol	-	-	-	-	-	-	-	-	-	-	-	27	Celiobiose	+	+	+	+	+	+	+	+	+	+	+
3	D-Arabinose	-	-	-	-	-	-	-	-	-	-	-	28	Maltose	+	+	+	-	+	-	+	+	+	+	+
4	L- Arabinose	-	-	-	+	-	+	-	-	-	-	-	29	Lactose	+	+	+	-	+	-	+	+	+	+	+
5	Ribose	+	+	+	+	+	+	+	+	+	+	+	30	Melibiose	-	+	+	-	+	-	-	+	+	-	+
6	D-Xylose	+	-	-	+	-	+	+	-	+	+	-	31	Sucrose	+	+	+	-	+	-	-	+	+	-	+
7	L-Xylose	-	-	-	-	-	-	-	-	-	-	-	32	Trehalose	+	+	+	-	+	-	+	+	+	+	+
8	Adonitol	-	-	-	-	-	-	-	-	-	-	-	33	Inulin	-	-	-	-	-	-	-	-	-	-	-
9	ß-Methyl-D-Xyloside	-	-	-	-	-	-	-	-	-	-	-	34	Melezitose	-	+	+	-	+	-	-	+	+	-	+
10	Galactose	+	+	+	+	+	+	+	+	+	+	+	35	Raffinose	-	+	-	-	+	-	-	+	+	-	-
11	Glucose	+	+	+	+	+	+	+	+	+	+	+	36	Starch	+	-	-	-	-	-	+	-	-	+	-
12	Fructose	+	+	+	+	+	+	+	+	+	+	+	37	Glycogen	-	-	-	-	-	-	-	-	-	-	-
13	Mannose	+	+	+	+	+	+	+	+	+	+	+	38	Xylitol	-	-	-	-	-	-	-	-	-	-	-
14	Sorbose	-	-	-	-	-	-	-	+	+	-	-	39	Gentibiose	+	+	+	+	+	+	+	+	+	+	+
15	Rhamnose	-	-	-	-	-	-	-	-	+	-	-	40	Turanose	-	-	-	-	-	-	-	+	+	-	-
16	Dulcitol	-	-	-	-	-	-	-	-	-	-	-	41	D-Lyxose	-	-	-	-	-	-	-	-	-	-	-
17	Inositol	-	-	-	-	-	-	-	-	+	-	-	42	D-Tagatose	-	-	-	+	-	+	-	-	+	-	-
18	Mannitol	+	+	+	+	+	+	-	+	+	-	+	43	D-Fucose	-	-	-	-	-	-	-	-	-	-	-
19	Sorbitol	-	-	-	-	-	-	-	+	+	-	-	44	L- Fucose	-	**-**	-	-	**-**	-	-	-	+	-	-
20	α-Methyl-D-Mannoside	-	-	-	-	-	-	-	+	+	-	-	45	D-Arabitol	-	**+**	+	-	**+**	-	-	-	-	-	+
21	α-Methyl-D-Glucoside	-	-	-	-	-	-	-	-	+	-	-	46	L- Arabitol	-	**-**	-	-	**-**	-	-	-	-	-	-
22	N-Acetyl-Glucosamine	+	+	+	+	+	+	+	+	+	+	+	47	Gluconate	+	**+**	-	-	**+**	-	+	+	+	+	-
23	Amygdalin	+	+	+	-	+	-	+	+	+	+	+	48	2-Keto-Gluconate	-	**-**	-	-	**-**	-	-	-	-	-	-
24	Arbutin	+	+	+	-	+	-	+	+	+	+	+	49	5-Keto-Gluconate	-	**-**	-	-	**-**	-	-	-	-	-	-
25	Esculin	+	+	+	+	+	+	+	+	+	+	+													

Gh1, C22, and C13: *Lactococcus lactis*; Com4, Pak1, Com5, C6, C7, and Pak7: *Lactobacillus plantarum*; Kp8 and Kp10: *Pediococcus acidilactici* (+ indicates utilization of sugars).

**Table 3:** Analysis of carbohydrate metabolism (ABI 50 CHL) and 16S rDNA sequence analysis (BLASTN) of BLIS-producing LAB isolates

**Sources**	**Strain**	**Species**	**% Accuracy (50 CHL )**	**% Similarity (BLASTN)**
Ghara	Gh1	*Lactococcus lactis*	99.9	98
Fresh curd (market)	Com4	*Lactobacillus plantarum*	99.6	97
	Com5	*Lactobacillus plantarum*	99.6	99
	Pak1	*Lactobacillus plantarum*	99.5	99
	Pak7	*Lactobacillus plantarum*	99.5	99
Dried curd	Kp8	*Pediococcus acidilactici*	99.6	95
	Kp10	*Pediococcus acidilactici*	99.6	99
Fermented cocoa beans	C22	*Lactococcus lactis*	99.6	99
	C6	*Lactobacillus plantarum*	99.9	98
	C7	*Lactobacillus plantarum*	99.9	98
	C13	*Lactococcus lactis*	99.6	99

### Identification of isolates by 16S rDNA sequencing

**Figure 1:**
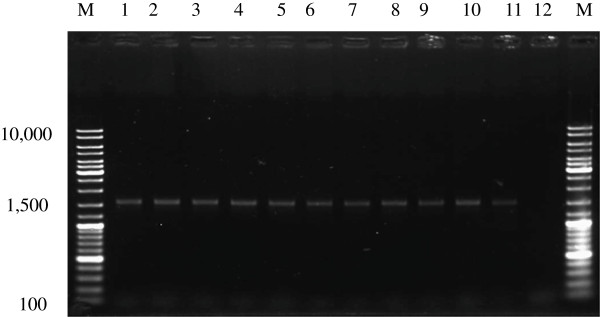
**Agarose gel electrophoresis of PCR products amplified using 16S rDNA primers.** M, GeneRuler DNA Ladder; lanes 1–11 are Kp8, Kp10, Gh1, Com4, Com5, Pak1, Pak7, C6, C7, C13, and C22, respectively; lane 12, negative control (no template).

PCR analysis of genomic DNA of the 11 isolates revealed bands that were the predicted size of 1.5 kb (Figure 1). The nucleotide sequences of these PCR products were compared with other 16S rDNA sequences in the GenBank database by BLASTN (Table 3).

### Phylogenetic analysis of isolate Kp10

**Figure 2:**
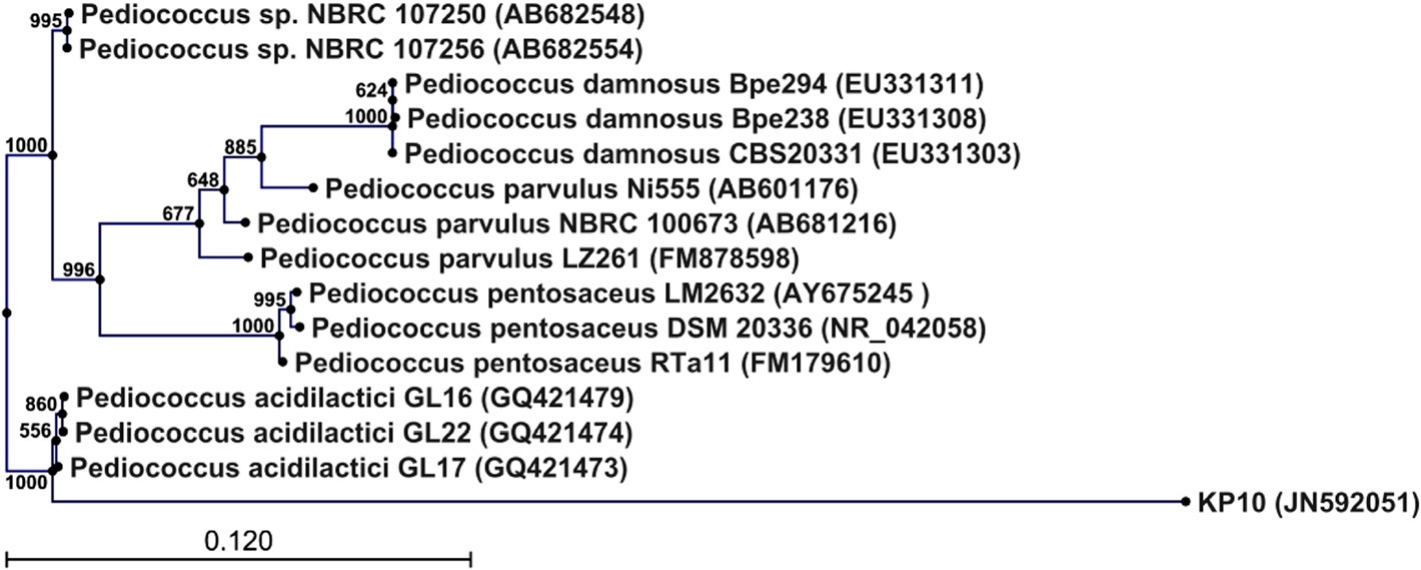
**Phylogenetic relationship of Kp10 with related species based on partial 16S rDNA gene sequence analysis.** The phylogenetic tree was constructed using the neighbour-joining method (CLC Sequence Viewer 6.5.2). The numbers at the nodes are bootstrap confidence levels (percentage) from 1,000 replicates. The scale bar represents 0.120 substitutions per nucleotide position. Reference sequences were obtained from the GenBank nucleotide sequence database.

A phylogenetic tree was generated by using the neighbour-joining method after aligning the nucleotide sequence of isolate Kp10 (accession number: JN592051) with sequences in the GenBank database (Figure 2). The isolate Kp10 formed a distinct cluster with *Pediococcus acidilactici,* supported by a bootstrap value of 100%.

### Physiological and biochemical characterization of isolate Kp10 (*P. acidilactici*)

The isolate Kp10 (*P. acidilactici*) was selected for further analysis based on its ability to produce high amounts of BLIS (Table 1). This bacterium was a gram-positive, catalase-negative coccus that was arranged in tetrads (Table 4). Kp10 demonstrated the ability to grow in the presence of 2% NaCl and within a temperature range of 30°C to 45°C.

**Table 4:** Characteristics of isolate Kp10

**Characteristics**	**Kp10 (*Pediococcus acidilactici*)**
Gram stain reaction	Gram-positive cocci
**Colony morphology**	
Size	>0.1 mm
Shape	Circular
Colour	Milky white
Elevation	Concave
Density	Mucoid and glistening
**Biochemical characteristics**	
Catalase	-
**Physiological characteristics**	
Growth in M17 broth:	
With 0.5% NaCl	+
With 2% NaCl	+
With 4% NaCl	-
With 6.5% NaCl	-
With 10% NaCl	-
At 5°C	-
At 10°C	-
At 30°C	+
At 35°C	+
At 37°C	+
At 45°C	+
At 60°C	-

Positive results (+), negative results (-).

As shown in Table 5, Kp10 (*P. acidilactici*) was susceptible to 18 antibiotics (penicillin G, erythromycin, ceftriaxone, amikacin, ciprofloxacin, norfloxacin, chloramphenicol, cefuroxime sodium, tetracycline, nalidixic acid, ampicillin, gentamycin, nitrofurantoin, sulfamethoxazole/trimethoprim, vancomycin, novobiocin, kanamycin, and oxytetracycline), and resistant to five antibiotics (lincomycin, colistin sulphate, bacitracin, polymixin B, and cefamandole).

**Table 5:** **Growth inhibition of *P. acidilactici *Kp10 by disc diffusion method**

**Antibiotic**		**Inhibition zone diameter**			
	**Disc content**	**Size (mm)**	**≤15 mm (R)**	**16–20 mm (I)**	**≥21 mm (S)**
Penicillin G	2 Units	24 (0)			+
Penicillin G	10 Units	26.5 (0.07)			+
Erythromycin	15 μg	32 (0)			+
Erythromycin	10 μg	30 (0)			+
Ceftriaxone	30 μg	33.08 (1.31)			+
Lincomycin	10 μg	0 (0)	+		
Colistin sulphate	10 μg	0 (0)	+		
Streptomycin	10 μg	18.63 (0.88)		+	
Amikacin	30 μg	24.83 (0.25)			+
Cloxacillin	5 μg	19 (0)		+	
Ciprofloxacin	10 μg	30 (0)			+
Norfloxacin	10 μg	24 (0)			+
Chloramphenicol	30 μg	32.28 (0.4)			+
Cefuroxime sodium	30 μg	34.25 (0.35)			+
Tetracycline	30 μg	29.5 (0.07)			+
Tetracycline	10 μg	24 (0)			+
Nalidixic acid	30 μg	31 (0)			+
Ampicillin	25 μg	32 (0)			+
Gentamycin	10 μg	22.5 (0.71)			+
Gentamycin	30 μg	28 (0)			+
Mecillinam	25 μg	19.72 (0.4)		+	
Nitrofurantoin	300 μg	30 (0)			+
Sulfamethoxazole/ trimethoprim	25 μg	31 (0.14)			+
Vancomycin	30 μg	24.75 (0.04)			+
Bacitracin	10 μg	0 (0)	+		
Novobiocin	30 μg	34.5 (0.07)			+
Kanamycin	30 μg	24.15 (0.21)			+
Neomycin	30 μg	20 (0)		+	
Polymixin B	300 Units	0 (0)	+		
Oxytetracycline	30 μg	21 (0)			+
Cefamandole	30 μg	12 (0)	+		

For all experiments coefficient of variation was ≤5 %. Results (zone of inhibition) are expressed as mean (SD).

R, resistant; I, intermediate; S, susceptible.

### β-galactosidase activity

The isolate Kp10 (*P. acidilactici*) produced blue/green colonies on M17 agar supplemented with X-gal and IPTG, which confirmed the ability to secrete β-galactosidase.

### Tolerance to bile salts

**Figure 3:**
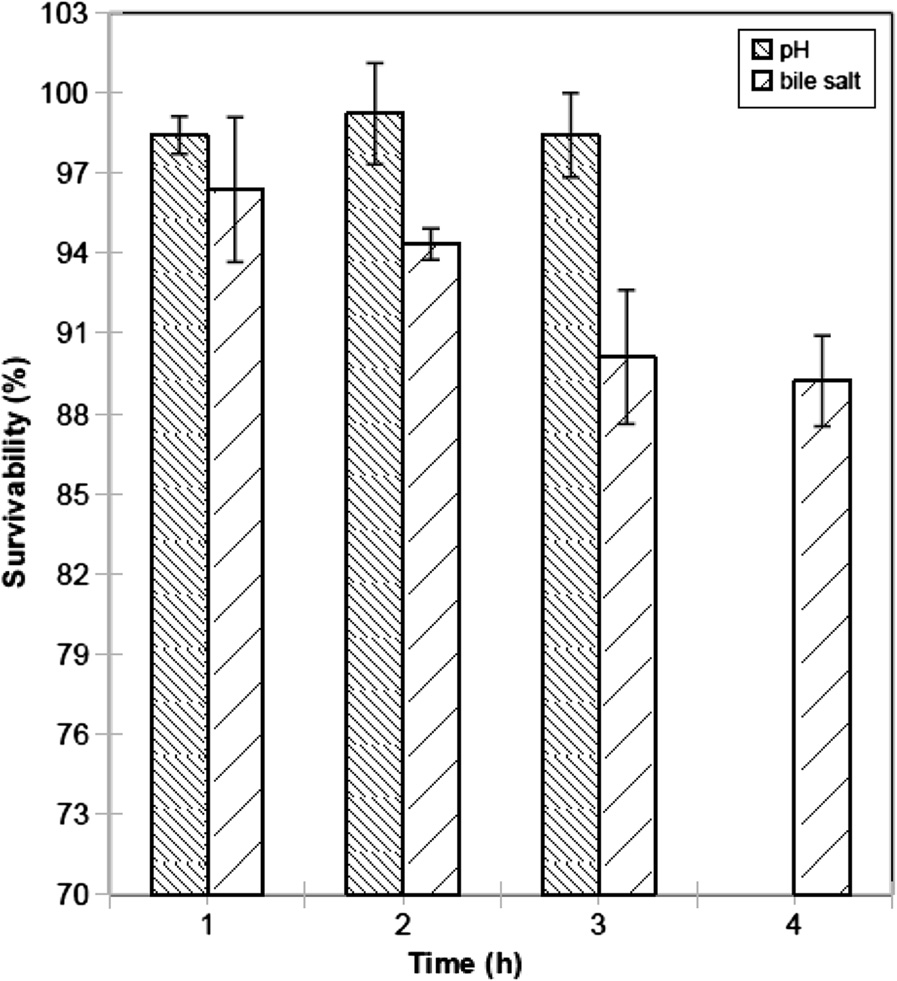
**Tolerance of the isolate Kp10 (*P. acidilactici*) to acidic conditions and bile salts.** Results are expressed as mean and standard deviation; tests were performed in triplicate.

The ability of Kp10 (*P. acidilactici*) to tolerate bile salts is shown in Figure 3. Percent survival was >95% after 1 h incubation but was reduced to 89% after 4 h.

### Tolerance to low pH

The ability of Kp10 (*P. acidilactici*) to tolerate acidic conditions is shown in Figure 3. Percent survival at pH 3 was >97% after 1 to 3 h incubation.

### Effect of pH and enzymes on BLIS activity

The effect of pH on Kp10 BLIS activity is shown in Table 6. BLIS was stable after a 1-h incubation at pH 2 to 9, but activity was considerably reduced at pH 10 and not detectable at pH 11. The effect of various enzymes on BLIS activity is shown in Table 7. Kp10 BLIS activity was retained in the presence of pepsin, α-amylase, and catalase but not in the presence of proteinase K or trypsin.

**Table 6:** Effect of pH on BLIS activity

**pH**	**BLIS activity (AU/ mL)**
Control	6,853
2	6,853
3	6,853
4	6,853
5	6,853
6	6,853
7	6,853
8	6,853
9	6,853
10	1,593
11	ND

ND, not detected.

**Table 7:** Effect of enzymes on BLIS activity

**Enzyme**	**BLIS activity (AU/mL)**
Control	6,853
Proteinase K	ND
Trypsin	ND
Pepsin	6,853
α-Amylase	6,853
Catalase	6,853

ND, not detected.

## Discussion and conclusions

In recent years much attention has focused on bacteriocin-producing LAB isolated from various sources, because bacteriocins are considered safe as food biopreservatives and can be degraded by gastrointestinal proteases [9]. However, LAB species present in traditional foods of Southeast Asian countries have not been widely studied [10]. In this study, 11 LAB strains isolated from traditional fermented milk products and cocoa beans from rural areas of Malaysia and Iran were found to produce antimicrobial substances. These LAB isolates were characterized, and two of the strains (Kp8 and Kp10) produced substances active against *Listeria monocytogenes* (888.56 AU/mL).

Phenotypic characterization based on sugar fermentation reveals biochemical properties of the microorganisms [11] but may not always provide a strong basis for LAB identification [12]. Although 16S rDNA sequence analysis is a powerful technique for identifying microorganisms and determining phylogenetic relationships [13], further analysis is needed for positive identification [14]. Therefore, we used both of these methods to identify the isolates. All 11 isolates were able to ferment ribose, galactose, glucose, fructose, mannose, n-acetyl-glucosamine, esculin, salicin, cellobiose and gentiobiose. Three different LAB species (*Lactococcus lactis*, *Lactobacillus plantarum*, and *Pediococcus acidilactici*) were identified using the API 50 CHL system and 16S rDNA analysis. Identification of Kp10 as *P. acidilactici* was confirmed by phylogenetic analysis (Figure 2).

In addition, β-galactosidase activity, tolerance to bile salts and acid conditions, and antimicrobial activity were to evaluate the probiotic properties of Kp10 (*P. acidilactici*). The isolate was able to grow in the presence of 2% NaCl, but growth was inhibited by 3% NaCl. Homofermentative LAB are more resistant than heterofermentative LAB to NaCl [15]. Pediococci strains are homofermentative, and tolerance to pH, temperature, and NaCl is species- and strain-dependent [16]. Bacterial cells cultured in high salt concentrations experience a loss of turgor pressure, which affects cell physiology, enzyme and water activities, and metabolism [17]; however, some bacteria overcome this effect by regulating osmotic pressure on both sides of the cell membrane [18]. Optimum temperature can also be used to differentiate among LAB strains [19]. Our results indicated that Kp10 (*P. acidilactici*) is a mesophile, which is in agreement with the findings of Ronald [20].

LAB are found in many natural environments; however, antibiotic resistance in these bacteria is a growing concern [21]. Thus, sensitivity to antibiotics must be determined before LAB strains can be used in food production [22]. Antibiotic-resistant strains can be detrimental to the health of humans and animals [21], because they are capable of transferring antibiotic resistance genes to pathogenic bacteria [23], which can contaminate raw food products such as meat or milk.

Data on the antibiotic susceptibility of *Pediococcus* spp. isolated from food are limited. Penicillin G, imipenem, gentamicin, netilmicin, erythromycin, clindamycin, rifampin, chloramphenicol, daptomycin, and ramoplanin are generally active against *Pediococcus* species [24-27]. However, susceptibility is thought to be species-dependent. We found that isolate Kp10 *(P. acidilactici*) was susceptible to ß-lactam antibiotics (penicillin G and ampicillin), as well as erythromycin, chloramphenicol, nitrofurantoin, and tetracycline. In contrast, previous studies have reported that LAB are often resistant to commonly used antibiotics such as β-lactams, cephalosporins, aminoglycosides, quinolone, imidazole, nitrofurantoin, and fluoroquinolones [23,28]. ß-lactams, which are bactericidal, are the most widely used class of antimicrobial agent because of their broad spectrum of action and excellent safety profile. ß-lactams inhibit bacteria cell wall synthesis and have a lethal effect on gram-positive bacteria. Erythromycin is a macrolide antibiotic with a range of action and efficacy similar to that of penicillin. Macrolides, which are bacteriostatic, bind to ribosomes to block protein synthesis and are effective against gram-positive microorganisms [29]. The rationale for this contradictory finding with those of Halami*, et al.*[28] and Herreros *et al.*[23] is not known. *Lactobacillus* and *Lactococcus* were previously reported to be susceptible to β-lactam antibiotics [29], which is in agreement with the findings of this study. It is possible that the reports of Halami *et al.* and Herreros *et al.* referred to LAB in general, whereas the present study specifically analyzed the species *P. acidilactici*.

The isolate Kp10 (*P. acidilactici*) was susceptible to a gram-negative antibiotic (nalidixic acid) and aminoglycosides (amikacin, kanamycin, neomycin, and streptomycin). In contrast, Zhou *et al.*[30] and Temmerman *et al.*[26] reported that most *Lactobacillus*, *Enterococcus*, and *Pediococcus* strains used as probiotics are resistant to gram-negative and aminoglycoside antibiotics. Thus, susceptibility to gram-negative antibiotics may be specific for this LAB species.

Vancomycin, an inhibitor of cell wall synthesis, is an important antibiotic because it is the last agent broadly effective against multi-drug resistant pathogens [29]. Kp10 (*P. acidilactici*) was not resistant to vancomycin, making it potentially useful for applications in the food industry [31]. Kp10 (*P. acidilactici*) was also susceptible to sulfonamide. Resistance to this antibiotic is caused by mutations in the gene encoding dihydropteroate synthase or by acquisition of plasmid-borne genes carrying sulfonamide-resistant forms of the enzyme [32].

Our results also showed that Kp10 (*P. acidilactici*) produced blue/green colonies when grown on M17 agar supplemented with X-gal and IPTG, demonstrating β-galactosidase activity. β-galactosidase is involved in lactose digestion and is used in the production of lactose-free milk. β-galactosidase–producing bacteria may also be potential probiotics to reduce lactose intolerance [33].

Mean bile concentration in the human gastrointestinal tract is 0.3% (w/v), with a residence time of about 4 h [34]. Therefore, we tested tolerance to bile salts at a concentration of 0.3%, which revealed 11% survival after 4 h. Bile salts interact with bacterial cell membranes, which are composed of lipids and fatty acids, inhibiting growth and killing many bacteria. The protonated (non-dissociated) form of bile salt exhibits toxicity by a mechanism similar to that of organic acids. This is involves intracellular acidification and collapse of the proton motive force, which in turn, inhibits the nutrient transport. However, some LAB strains are able to hydrolyze bile salts with bile salt hydrolase [35].

Resistance to low pH is one of the major criteria for selecting strains for probiotic applications [36]. Survival of Kp10 (*P. acidilactici*) at pH 3 exceeded 97%, suggesting its potential for use as a probiotic.

Several strains of *P. acidilactici* isolated from the intestine of healthy dairy cows and characterized using methods similar to those used in the present study were found to inhibit *Escherichia coli*. [37]. The authors reported that *P. acidilactici* was resistant to acid and bile salts, indicting the ability to survive and colonize in the intestine. In the present study, we found that Kp10 (*P. acidilactici*) was active against the pathogen *L. monocytogenes.* It is interesting to note that *P. acidilactici* from two different agricultural sources (intestine of dairy cows and a traditional milk product) showed promising prophylactic properties.

We found that the BLIS from Kp10 (*P. acidilactici*) was stable in a wide range of pH (2–9), suggesting that its antimicrobial activity was not due to the pH of the cell-free supernatant. The reduced activity at high pH was probably due to denaturation of the protein. A similar result was also observed for an antimicrobial compound produced by *Lactococcus lactis*, which was active at the pH range 2 to 10 and completely inactivated at pH 12 [38].

Since bacteriocins are proteinaceous substances, they must be sensitive to at least one proteolytic enzyme [39]. Therefore, bacteriocins can be identified in part by exposure to proteolytic enzymes [40]. We found proteolytic enzyme treatment reduced the activity of the antimicrobial compound secreted by Kp10 (*P. acidilactici*). However, activity was not reduced by catalase, indicating that H_2_O_2_ was not responsible for microbial inhibition, or α-amylase activity, indicating that the compound was not glycosylated, which is characteristic of most bacteriocins [41]. Complete inactivation activity was observed after treatment with proteinase K and trypsin, in accordance with a report by Albano *et al.*[42] of pediocin PA-1 activity [43]. Treatment with pepsin did not alter the antimicrobial activity of the BLIS in this study; however, proteolytic enzymes do not always reduce the antimicrobial activity of a bacteriocin [44]. Stability in the presence of a proteolytic enzyme could be due to unusual amino acids in the bacteriocin structure or cyclic N-terminal or C-terminal protected peptides [45].

We conclude that isolate Kp10 (*P. acidilactici*) is a potential probiotic that may exert beneficial positive effects on intestinal flora, because the strain is tolerant of bile salts (0.3%) and acidic conditions (pH 3). To better understand its potential as a probiotic, future studies are needed to characterize the interactions of this *P. acidilactici* strain to the intestinal mucosal epithelium.

## Methods

### Isolation of lactic acid bacteria

Fresh curds (three varieties), dried curds (four varieties), ghara (one variety), and fermented cocoa beans were obtained from family-owned businesses in rural areas of Malaysia and Iran. Ghara is a traditional flavor enhancer that is popular in northern Iran. It is prepared by incubating a blend of flour with whey at room temperature for several days. Food samples (25 mL or 25 g, depending on type of sample) were mixed with 225 mL de Man Rogosa Sharpe (MRS) medium (Merck, Darmstadt, Germany). After a 24-h incubation at 30°C, cultures were serially diluted (10-fold) in buffered Andrade peptone water (BioChemika, India). To prepare agar plates, MRS and M17 agar (Merck, Darmstadt, Germany) were supplemented with 0.01% (w/v) sodium azide to inhibit the growth of gram-negative bacteria. Diluted samples (100 μL) were spread on agar plates and incubated in anaerobic conditions at 30°C for 24 to 72 h. The isolates were evaluated by cell morphology, Gram stain reaction, and biochemical and physiological characteristics.

### Physiological and biochemical characterization

#### Cell morphology and Gram stain

Gram staining was carried out according to the routine procedure, and cell morphology was examined by light microscopy.

#### Catalase activity

Catalase activity was determined by adding a drop of 3% (v/v) H_2_O_2_ on a colony. Immediate effervescence was indicated a positive reaction.

#### Glucose fermentation test

Nutrient agar was prepared with 1% (w/v) of glucose and 0.004% (w/v) bromocresol purple (Sigma) as a pH indicator. Cultures (10 μL) were spread on the prepared agar. A yellow zone around the culture after 24-h incubation at 37°C indicated acid production.

#### Effect of NaCl concentration on growth

The isolates were inoculated (1% v/v) into M17 broth containing different concentrations of NaCl (0.5%, 2%, 4%, 6.5%, or 10% [w/v]) and bromocresol purple and incubated at 37°C. After 48 h, growth was evaluated, indicated by a color change from purple to yellow.

#### Effect of temperature on growth

The isolates were inoculated (1% v/v) into M17 broth containing bromocresol purple and incubated for 48 h at different temperatures (4°C, 10°C, 30°C, 35°C, 37°C, 45°C, or 60°C). During the incubation, growth was evaluated at time intervals, indicated as a color change from purple to yellow.

#### Effect of low pH on growth

The isolates (1 mL) were inoculated into 9 mL sterile M17 broth, and the pH was adjusted to 3 using 0.5 N HCl. During incubation, growth was monitored as optical density at 650 nm using a spectrophotometer (Perkin Elmer, Lambda 25, USA). After incubation for 0, 1, 2, 3, or 4 h, viable microorganisms were enumerated using the pour plate technique. Diluted cultures (100 μL) were mixed with cooled M17 agar, poured into plates, and incubated at 37°C for 24 to 48 h. The number of colonies was determined using a colony counter and compared with the control (0 h) to determine acid tolerance [46].

Percent survival was calculated as follows:

(1) $$\text{Percent survival}=\left({\text{log cfu t}}_{\text{f}}/{\text{log cfu t}}_{\text{i}}\right)\times 100\%$$

where t_f_ is the incubation time and t_i_ is 0 h (control).

#### Effect of bile salts on growth

Bile tolerance of the isolates was determined by the viable count method [47]. The isolates (1 mL) were inoculated into 9 mL sterile M17 broth enriched with 0.3% (w/v) bile salts (Oxoid) and incubated at 37°C. Growth was monitored as optical density at 650 nm using a spectrophotometer. After incubation for 0, 1, 2, 3, or 4 h, viable microorganisms were enumerated using the pour plate technique. The number of colonies was determined using a colony counter and compared with the control (0 h) to determine bile salt tolerance. Percent survival was calculated using Equation 1.

#### Antibacterial susceptibility testing

Susceptibility to 24 antibiotics was determined by using the disc diffusion method [48]. Single colonies were inoculated into M17 broth and incubated at 37°C for 24 h. A sterile cotton wool swab dipped into the bacterial suspension was used to spread bacteria evenly on the surface of M17 agar plate.

Commercially available antibiotics discs (Oxide) containing penicillin G (2 units), erythromycin (10 μg), ceftriaxone (30 μg), colistin sulphate (10 μg), streptomycin (10 μg), amikacin (30 μg), norfloxacin (10 μg), chloramphenicol (30 μg), tetracycline (10 μg), nalidixic acid (30 μg), ampicillin (25 μg), gentamycin (30 μg), mecillinam (25 μg), nitrofurantoin (300 μg), sulfamethoxazole/trimethoprim (25 μg), vancomycin (30 μg), kanamycin (30 μg), neomycin (30 μg), lincomycin (10 μg), cloxacillin (5 μg), ciprofloxacin (10 μg), cefuroxime sodium (30 μg), bacitracin (10 μg), or novobiocin (30 μg) were carefully placed on the surface of the dried agar plates to ensure uniform contact between the disc and agar. The plates were then incubated at 30°C for 24 h.

Inhibition zones (including the disc diameter) were measured, and isolates were categorized as sensitive (≥ 21 mm), intermediate (16–20 mm), or resistant (≤ 15 mm), as previously described [29,49].

#### β-galactosidase activity

The method described by Karasova *et al.*[50] was used to test for β-galactosidase activity. The isolate was incubated at 37°C for 24 h on an MRS agar plate containing 0.01% X-gal (5-bromo-4-chloro-3-indolyl β-D-galactopyranoside, Vivantis, Malaysia) and 0.1 mM IPTG (isopropyl β-D-1-thiogalactopyranoside, Vivantis) dissolved in dimethyl sulfoxide.

#### Identification of isolates using API 50 CHL

API 50 CHL strips (API systems, bioMérieux, France) were used to characterize the isolates, according to the manufacturer’s instructions. The inoculated strips were incubated at 30°C, and the reactions were observed after 48 h. The API database (bioMérieux SA) and accompanying computer software were used to interpret the results. Readings were taken after a 48-h incubation at 30°C. Growth on a particular substrate changed the color of the medium from violet to yellow, which was scored on a 5-point scale (intense yellow = 5). A score ≥3 was considered a positive result. The test was performed in triplicate.

#### Identification of isolates by 16S rDNA sequencing and phylogenetic analysis

The isolates were identified by 16S rDNA sequencing to confirm the results obtained from biochemical identification. Briefly, the procedure is as follows.

##### DNA extraction

DNA was extracted using the method described by Leenhouts *et al.*[51], with some modifications. Cells harvested from an overnight culture (1.5 mL) were resuspended in 200 μL distilled water containing 12 mg/mL lysozyme and incubated at 37°C for 90 min. After adding 100 μL sodium dodecyl sulfate (15% (w/v), the solution was mixed by gentle inversion and incubated at 65°C for 5 to 10 min until the mixture was clear. Ice-cold 3 M sodium acetate (300 μL, pH 5.2) was added, and the solution was mixed gently, incubated on ice for 10 min, centrifuged at 15,000 × *g* for 12 min at 4°C, and then transferred to another tube. Phenol (600 μL) was then added, and the solution was centrifuged for 12 min at 15,000 *× g* at room temperature. The upper layer containing DNA was transferred to a clean tube, and the DNA was precipitated by incubation at −20°C overnight with one volume of 3 M sodium acetate and two volumes of ice-cold isopropanol. After centrifugation at 15,000 *× g* at 4°C for 10 min, the supernatant was carefully removed by pipetting, and the DNA pellet was washed with 1 mL ice-cold ethanol (70% v/v). To remove the alcohol, the sample was centrifuged at 15,000 × *g* for 10 min. The DNA was air-dried for 15 to 30 min before adding 40 μL 1× Tris-EDTA buffer and 2 μL RNase and then incubated at 37°C for 15 min. The DNA was stored at −20°C for subsequent use in experiments.

The DNA was analyzed by 0.7% (w/v) agarose gel electrophoresis at a constant voltage of 75 V for 45 min until the methylene blue dye reached approximately 10 mm from the base of the gel.

##### Sequencing and phylogenetic analysis

The isolates were identified by PCR analysis using a set of primers (27 F and 1542–1522 R) specific for bacterial 16S rDNA [52] according to the method described by Chong *et al.*[53], with a slight modification. Briefly, for hot-start PCR, the polymerase was activated at 95°C for 5 min. PCR was performed as follows: denaturing at 95°C for 1 min, annealing at 55°C for 1 min, and extension at 72°C for 1 min for 30 cycles, followed by a final extension step at 72°C for 10 min.

After agarose gel electrophoresis, the PCR products were purified using the Wizard SV Gel and PCR Clean Up Kit (Promega, Madison, WI, USA) according to the manufacturer’s instructions. The PCR products were sequenced and compared with reference sequences by conducting a BLAST search of the GenBank database (http://www.ncbi.nlm.nih.gov/blast/Blast.cgi). The 16S rDNA sequences were aligned using CLC Sequence Viewer 6.5.2, and a phylogenetic tree was constructed using the neighbour-joining method. Bootstrap resampling was carried out with 1,000 replications to estimate the confidence of tree topologies.

#### Antimicrobial activity test

The antimicrobial activity of the isolates was determined by the agar well diffusion method [54] using cell-free culture supernatants. The isolates were grown in M17 broth at 30°C for 24 h, and the cultures were centrifuged at 12,000 × *g* for 20 min at 4°C (rotor model 1189, Universal 22R centrifuge, Hettich AG, Switzerland). The supernatant (100 μL) was placed into 6-mm wells of agar plates that were previously seeded (1% v/v) with the actively growing test strain. The plates were incubated under optimal conditions for growth of the target microorganism. After 24 h, the growth inhibition zones were measured, and antimicrobial activity (AU/mL) was determined as described by Parente *et al.*[55].

#### Effect of pH and enzymes on BLIS activity

The effect of pH on BLIS activity in the cell-free culture supernatant was evaluated by adjusting the pH from 2 to 11 with 1 N HCl or 1 N NaOH [41]. The cell-free culture supernatant was incubated at 37°C for 1 h before measuring BLIS activity. Sensitivity to enzymes was determined after a 2-h incubation with proteinase K, trypsin, pepsin, α-amylase, and catalase (final concentrations, 1 and 0.1 mg/mL) (all obtained from Sigma). The samples were incubated at 37°C, except for samples containing trypsin and catalase, which were incubated at 25°C and 37°C.

## Competing interests

The authors declare that they have no competing interests.

## Authors’ contributions

SA carried out all the experimental work, which include strains isolation and characterization as well as identification of the antimicrobial substances, and also drafted the manuscript. JST conceived of the study and participated in experimental design. All authors contributed to the design and interpretation of experimental results, as well as editing and revising the manuscript. All authors have read and approved the final manuscript.
